# Supplementary material for: InGaN Nanorods Decorated with Au Nanoparticles for Enhanced Water Splitting Based on Surface Plasmon Resonance Effects
Source: Nanomaterials (Basel). 2020 May 9;10(5):912. doi: 10.3390/nano10050912 (PMC7279278; doi:10.3390/nano10050912)
Supplement: Supplementary file 1 [file nanomaterials-10-00912-s001.pdf]

Article

InGaN Nanorods Decorated with Au Nanoparticles for Enhanced Water Splitting Based on Surface Plasmon Resonance Effects

Qing Liu [1], Jiang Shi [1], Zhenzhu Xu [2], Bolin Zhang [1], Hongliang Liu [1], Yinlei Lin [3], Fangliang Gao [1,*], Shuti Li [1] and Guoqiang Li [2,*]

1 Guangdong Engineering Research Center of Optoelectronic Functional Materials and Devices, Institute of Semiconductors, South China Normal University, Guangzhou 510631, China; 2018022795@m.scnu.edu.cn (Q.L.); 2018022807@m.scnu.edu.cn (J.S.); berlinzhang@foxmail.com (B.Z.); 2019022815@m.scnu.edu.cn (H.L.); lishuti@scnu.edu.cn (S.L.)

2 State Key Laboratory of Luminescent Materials and Devices, South China University of Technology, Guangzhou 510640, China; zhenzhupearl@163.com

3 School of Materials Science and Energy Engineering, Foshan University, Foshan 528000, China.; linyinlei@fosu.edu.cn

* Correspondence: gaofl@m.scnu.edu.cn (F.G.); msgli@scut.edu.cn (G.L.)

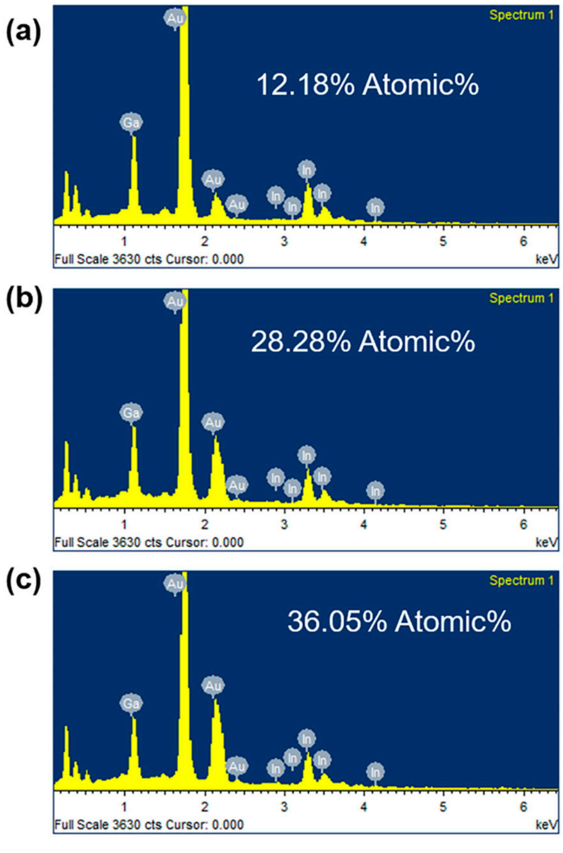

Figure S1. EDS images of Au5@NRs, Au10@NRs, Au15@NRs.
